# VIRTUAL REALITY MEDICAL EDUCATION PROJECT ENHANCES EMPATHY

**DOI:** 10.1093/geroni/igz038.1096

**Published:** 2019-11-08

**Authors:** Marilyn R Gugliucci

**Affiliations:** University of New England College of Osteopathic Medicine, Biddeford, Maine, United States

## Abstract

Introduction: It is particularly important that innovative learning modalities are utilized to augment medical students’ learning about empathy in relation to older adult health care. As the older population increases and lives longer, their health care utilization is predicted to increase dramatically. Methods: 1st year osteopathic medical students (N=174) at the University of New England were required to complete the National Network of Libraries of Medicine (NN/LM) New England Region (NER) grant funded Embodied Labs’ “We Are Alfred” Virtual Reality (VR) module (15 min) and a pre/post-test. The students assumed the role of Alfred, a 74 y/o African American male with macular degeneration and hearing loss. “We Are Alfred” utilizes a virtual reality headset, headphones, and a hand-tracking device to immerse students into Alfred’s experiences as a patient. Descriptive statistics and t-tests were applied for data analyses. Results: Learning was broad and significant: 94% reported increased empathy; 92% reported increased learning about macular degeneration; and 90% reported increased learning about hearing loss. Qualitative data collected from the pre-tests and post-tests supported learning on empathy with 4 associated themes (Personal Experiences, Perceptions of Older Adults, Thoughts about Health, Descriptors of Aging).. Conclusion: Virtual reality was deemed a successful medical education learning tool for these medical students. Utilizing this technology to create an immersive case study taught these medical students about the aging experience from the first-person patient perspective.

